# A holistic evaluation of nitrogen responses in maize

**DOI:** 10.1093/plphys/kiae029

**Published:** 2024-01-20

**Authors:** Maria-Angelica Sanclemente

**Affiliations:** Assistant Features Editor, Plant Physiology, American Society of Plant Biologists; Horticultural Sciences, University of Florida, Gainesville, FL, USA

Nitrogen (N) fertilization has been one of the main practices used to increase yield of agricultural crops worldwide. In developed countries, N supplementation in agriculture has increased by more than 120% between the 1960s and 2020 (Adalibieke et al. 2023). It is estimated that N applications will continue to rise as world population is expected to grow by 3 billion people within the next 80 years (Verma et al. 2023). Moreover, a 56% increase in crop yield will be needed to sustain the predicted population growth (Verma et al. 2023). However, pollution by excess N runoff from agriculture remains a global concern (de Vries 2021). A holistic approach is thus needed to integrate knowledge of plant nitrogen use efficiency with management practices.

Toward this end, understanding plant responses to N and the associated mechanisms for uptake and utilization is central to developing more sustainable fertilization strategies in a crop-specific manner. For instance, in maize, physiological responses to N supplementation varies within hybrids (Ige et al. 2021; Kosola et al. 2023). Understanding N responses at the cellular, molecular, genetic, and whole-plant levels can not only lead to improved fertilizer applications, but also improve breeding for better yields.

In this issue of *Plant Physiology*, Ying et al. (2023) conducted a multilevel assessment of physiological effects of N supplementations on different maize hybrids. The authors selected 5 lines including foundational breeding germplasm from public and private sectors including commercial agricultural hybrids such as B73xMo17, B73xPHN82, B73xPHZ51, PHB47xPHN82, and LH195xPHN82. The conditions tested mimicked commercial growing conditions in the state of Michigan so that results presented there could have a wide impact on maize-growing areas in the United States and abroad.

All the hybrids used in this study were responsive to N, and a lack of supplementation was sensed as a stress. However, the magnitude of these responses varied greatly among hybrids. For instance, fertilization with 122 pounds of N per acre tripled yield of PHB47xPHN82 from 60 bushels/acre in low N to 173 bushels/acre in the high N treatment. In contrast, B73xPHZ51 hybrid yield increased by only about 20% in response to N. Similarly, across all hybrids, grain moisture increased in high N (Fig. 1A), and the silking to anthesis interval (ASI) was reduced. These results suggest that N supplementation extends grain maturation and vegetative states while improving pollination.

**Figure 1.**
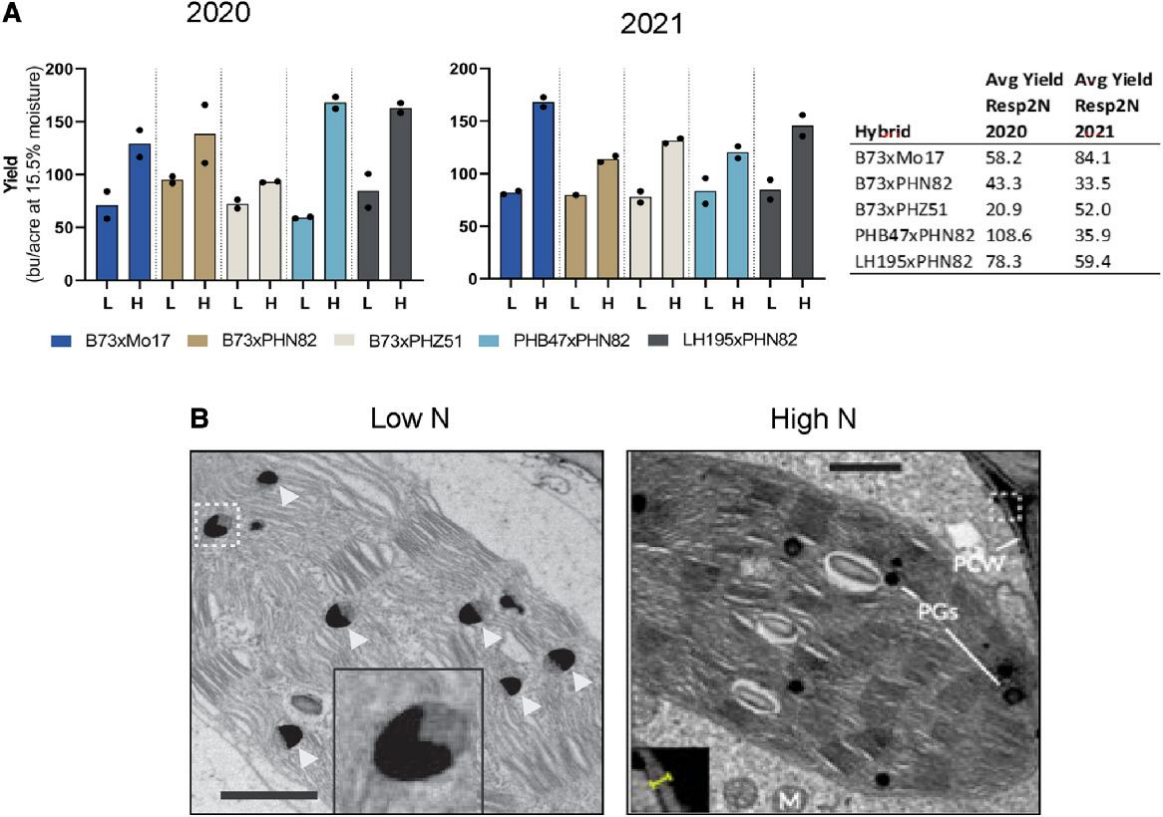
Physiological responses to N in different maize hybrids. **A)** Hybrid moisture-adjusted yield in 2 growing seasons between 2020 and 2021. The table indicates the calculated response to N (Resp2N) in each hybrid. **B)** Representative images of plastoglobule morphology in the mesophyll of plants growing in low N (left) and supplemented N (right). Triangles indicate crescent-shaped plastoglobuli in low N. PG, plastogluble; PCW, primary cell wall; M, mesophyll. Adapted from Ying et al. (2023), Fig. 5C and Fig. 8, A to C.

Consistent with the above findings, nitrogen fertilization increased levels of chlorophyll precursors and prenyl-lipid compounds, indicating that photosynthetic active tissues are retained longer. The response was observed across all hybrids, with more significant effects in the B73xMo17 hybrid. In contrast, high N reduced levels of phenolics throughout development. Although this response was shared across all hybrids, effects on individual phenolic compounds showed a hybrid and developmental component. For example, *p*-coumeric acid was significantly elevated in response to N in B73xPHZ51 kernels in the R2 stage (10–12 days after pollination), but it was significantly reduced in B73xPHN82 seeds.

The retention of photosynthetic activity and high prenyl-lipid compounds in response to N is consistent with changes in cell morphology observed in all hybrids. Low N increased cell wall thickness, whereas high N reduced it. Moreover, N altered chloroplast components such as oil-containing bodies known as plastoglobules (Fig. 1B). These structures represent an important carbon (C) sink through lipids and a storage site for prenyl-lipid compounds, so changes in their morphology and composition can be used as markers for plant stress and developmental changes (Austin et al. 2006; Arzac et al. 2022). Accordingly, hybrids in low N conditions, which senesced earlier, had crescent-shaped plastoglobules, whereas plants in high N had smaller and fewer plastoglobules (Fig. 1B). Although changes in cell morphology were not statistically significant among hybrids due to the low numbers of biological replicates, the results add another level of understanding to N responses and C/N dynamics in maize.

Differential expression analysis identified 8 genes that were differentially expressed among all hybrids and across all developmental stages. These genes then provide an important set of markers for N status in maize. However, the physiological significance of some of these genes remains unclear, such as genes involved in thiamine biosynthesis, a response previously observed in maize in response to N. Profiles of other genes were consistent with metabolic and structural responses, such as *Carotenoid Cleavage Dioxygenase 4*, *ZmCC4* in high N. *ZmCC4* is negatively associated with senescence and positively associated with prenyl-lipid metabolism during advanced stages of development.

The work of Ying et al. (2023) is notable as it integrates molecular, genetic, metabolic, developmental, structural, and grain-quality parameters in commercial growing conditions. The work suggests new players in the nitrogen response of maize and highlights the importance of data analysis to identify genes of interest in the context of plant and seed development. The high variability among hybrid responses to N indicates different strategies used by these lines to maximize growth and yield during N abundance and supporting the importance of customized fertilization programs for more sustainable production. This comprehensive investigation of N effects on maize opens the door for similar studies in other crops.

## Data Availability

Data presented here is available in Ying et al. (2023), https://doi.org/10.1093/plphys/kiad583.

## Dive Curated Terms

The following phenotypic, genotypic, and functional terms are of significance to the work described in this paper:

nitrogen CHEBI: CHEBI:25555
